# From metabolism to genome integrity: NRF2 as a key mediator of cancer therapy response

**DOI:** 10.3389/fcell.2026.1868571

**Published:** 2026-07-03

**Authors:** Andrea Sarrecchia, Claudia Di Girolamo, Irene Taddei, Claudia Contadini, Daniela Barilà, Claudia Cirotti

**Affiliations:** 1 Department of Biology, PhD Program in Cellular and Molecular Biology, University of Rome “Tor Vergata”, Rome, Italy; 2 Laboratory of Cell Signalling, IRCCS-Fondazione Santa Lucia, Rome, Italy; 3 Department of Biology, University of Rome “Tor Vergata”, Rome, Italy; 4 Preclinical Models and New Therapeutic Agents Unit, IRCCS Regina Elena National Cancer Institute, Rome, Italy

**Keywords:** chromatin accessibility, DNA damage response, immune evasion, metabolic rewiring, therapy resistance

## Abstract

Metabolic rewiring in cancer is sustained by deregulated intracellular signaling, frequently due to genetic mutation of oncogenes, tumor suppressors or oncometabolite genes. These mutations strongly affect the activity of Transcription Factors (TFs) and epigenetic remodelling factors, ultimately leading to different metabolic subtypes. Of note, several metabolites have been proposed to significantly modulate gene expression through post-translational modifications of metabolic enzymes, TFs and histones, sustaining the intracellular feedback loop in cancer cells. Among the TFs involved in metabolic reprogramming of cancer cells, NRF2, a well-characterized factor in the oxidative stress response, emerged as a crucial player. NRF2 is at the crossroads of critical intracellular pathways linking metabolism, DNA damage and chromatin remodelling. These findings point to NRF2 as a pivotal player in cancer therapy resistance**,** making it a promising target to ameliorate patient survival. Remarkably, high expression and transcriptional activity of NRF2 have been demonstrated in several cancer types, where its expression and activity correlate with poor patient survival. Unfortunately, direct molecular targeting of TFs is still challenging. Therefore, identifying signaling pathways that either drive NRF2 deregulation or are sustained by its hyperactivation has emerged as an alternative strategy to enhance NRF2 targetability and ultimately improve therapeutic outcomes. Here, we aim to summarize the current knowledge on the role of NRF2 in resistance to cancer therapy, focusing on its regulation of gene expression, metabolism, and immune response in cancer, while exploring potential functional links between metabolic rewiring, chromatin remodelling and DNA damage response proficiency.

## 1 Introduction

Nuclear factor (Erythroid-derived 2)-like 2, NRF2, is a transcription factor belonging to the Cap’n Collar (CNC) subfamily, a group of basic leucine zipper (bZIP) transcription factors, well-known in oxidative stress response. It is encoded by the *NFE2L2* gene, and it contains seven specific functional domains called Neh1-7, among which Neh1 ensures the DNA recognition and binding. The Neh2 domain is responsible for NRF2 targeted degradation: in homeostatic conditions NRF2 levels are low and tightly controlled by its cytosolic repressor, the Kelch-like ECH-associated protein 1 (KEAP1), which binds two motifs on NRF2 Neh2, DLG and ETGE. This binding leads to the subsequent ubiquitination of NRF2 by Cullin-3 RING ubiquitin ligase and its consequent proteasomal degradation (Tong et al., 2006). Reactive oxygen species (ROS) and other electrophiles can be sensed by KEAP1 causing its conformational change. In this state, according to the “Hinge and latch” model, newly synthetized NRF2 can escape KEAP1 trap, translocate in the nucleus and mediate the transcription of its target genes (Horie et al., 2021).

To this matter, NRF2 binds its consensus sequences, known as Antioxidant Response Elements (ARE), whose core sequence is 5′-*GTGANNNGC*-3′ but varies consistently between different loci. This structural divergence of ARE variants, alongside their partial homology with related response elements (such as AP-1), fosters a competitive environment between several transcription factors (reviewed in (Raghunath et al., 2018)). This regulatory landscape of ARE sequences therefore necessitates nuanced understanding of how binding affinity and motif context dictate gene-specific induction, elucidating the distinct contributions of specific co-activators or repressors to the NRF2-driven transcriptional program.

Importantly, this regulatory efficiency becomes a double-edged sword in the context of malignancy. Indeed, despite its relevance in orchestrating antioxidant defences and drug detoxification which would address it as a tumor suppressor protein, NRF2 is constitutively stabilized and hyperactivated in certain tumors, such as non-small cell lung carcinoma (NSCLC) and glioblastoma (GB), contributing to tumor growth and therapy resistance (Pölönen et al., 2019; Cloer et al., 2019; Zhang, 2025; Frank et al., 2018). Both genetic alteration or non-genomic causes may drive NRF2 aberrant activation in cancer. NRF2 can be overexpressed due to copy number amplification, *NFE2L2* gene promoter demethylation or oncogene-dependent increased transcription. In addition, loss of function mutations of *KEAP1*, and gain of function mutations of *NFE2L2* genes, or mutations/deletions in *CUL3*, much rarer and less studied, are frequently observed (Cloer et al., 2019; Zhang, 2025; Frank et al., 2018; Padmanabhan et al., 2006; DeNicola et al., 2011; Martinez et al., 2015). The clinical relevance of this crosstalk is highlighted by the frequent occurrence of somatic NRF2 mutations in human cancers, where mutations in the DLG and ETGE motifs disrupt KEAP1-mediated degradation and lead to constitutive NRF2 activation and poor prognosis (Shibata et al., 2008).

Post-translational modifications (PTMs) are also important modulators of NRF2 functionality and signaling: phosphorylation, acetylation, ubiquitination can significantly affect NRF2 protein stability, localization and activity (Yang et al., 2025). Protein-protein interaction represents another important level of NRF2-pathway regulation acting as competitive-binding proteins that prevent NRF2-KEAP1 association. As an example, p62 can sequester KEAP1 and promote NRF2 stabilization, while p21 has been reported to directly bind NRF2 and promote its activation (Cloer et al., 2019; Ichimura et al., 2013; Chen et al., 2009).

A critical question regarding NRF2 deregulation in cancer is how the signal transduction networks activated downstream of NRF2 contribute to tumor progression, aggressiveness, and resistance to therapy. NRF2 role in the modulation of metabolic rewiring, chromatin accessibility, DNA repair proficiency and inflammation will be discussed, lastly tackling therapy resistance as a direct consequence of these alterations.

### 1.1 NRF2: metabolic gene expression, acetylation and chromatin accessibility

Metabolic adaptation of cancer cells dates to 1920s when Warburg effect was first described. Cancer cells preferentially rely on aerobic glycolysis, converting glucose into lactate while maintaining high glycolytic flux to fuel anabolic pathways (Koppenol et al., 2011). NRF2 emerges as a key regulator of metabolic rewiring, orchestrating the transcription of genes involved in redox homeostasis and metabolism, and contributing to the maintenance of anabolic pathways and metabolites availability, ultimately linking metabolic reprogramming to epigenetic regulation and chromatin remodelling (Hayes and Dinkova-Kostova, 2014; Xi et al., 2023; Okazaki et al., 2020). Interestingly, NRF2 redirects glucose and glutamine toward anabolic pathways in lung cancer cells, modulating purine nucleotide biosynthesis and glutamine metabolism (Mitsuishi et al., 2012). NRF2 signaling induction has been linked to the hyperactivation of PI3K-AKT pathway, frequently observed in those tumors having PTEN alteration. Indeed, NRF2 acts as a downstream effector of PI3K-AKT pathway (Mitsuishi et al., 2012) and pharmacological inhibition of this pathway reduces NRF2 nuclear accumulation (Harrison et al., 2006; So et al., 2006). Accordingly, we found that PTEN-null GB cells exhibit constitutive activation of the NRF2 pathway downstream of SRC tyrosine kinase activity and subsequent PI3K-AKT-mTORC1 induced signaling (Cirotti et al., 2024). More recently, we identified a critical role for NRF2 in sustaining GB metabolism (Cir et al., 2025). We demonstrated that Caspase-8 expression and its SRC-dependent phosphorylation on Tyrosine 380 (Y380) drive NRF2 hyperactivation, identifying NRF2 as the final effector of SRC-Caspase-8-mTORC1 axis supporting cellular respiration and bioenergetics (Cir et al., 2025). Constitutive NRF2 activation has been observed also in esophageal squamous cell carcinoma (ESCC) harbouring Caspase-8 mutations (Li et al., 2022). Similarly to SRC-dependent Caspase-8 phosphorylation on Y380, Caspase-8 mutants sustain mTORC1 signaling, leading to p62 phosphorylation and KEAP1 sequestration, ultimately resulting in NRF2 activation (Cir et al., 2025; Li et al., 2022). Overall, these studies highlight the Caspase-8-NRF2 axis as a novel prognostic factor in cancer, where NRF2 can contribute to enhanced resistance to exogenous stresses, including chemotherapy and radiotherapy.

NRF2-mediated metabolic rewiring in cancer also influences the availability of metabolites, including α-ketoglutarate (α-KG), nicotinamide adenine dinucleotide (NAD) and acetyl-Coenzyme A (acetyl-CoA), that have been extensively shown to profoundly influence gene expression in cancer by affecting the activity of epigenetic enzymes, TFs, and histone proteins, ultimately shaping chromatin accessibility (Wang et al., 2024). These observations indicate that NRF2 directly and indirectly remodels chromatin accessibility through the metabolic control. Among metabolites, acetyl-CoA links energy status to transcriptional control. Indeed, beyond its role in the TCA cycle and lipid biosynthesis, it provides the primary donor for histone acetylation, directly impacting gene expression (Takah et al., 2006). Notably, NRF2-driven metabolic rewiring can influence acetyl-CoA availability, establishing a functional link between oncogenic signaling, metabolic adaptation, and epigenetic regulation. In this regard, *NRF2* promotes hepatocellular carcinoma (HCC) progression, sustaining acetyl-CoA production thereby influencing gene expression programs (Xi et al., 2023). Consistently, *NRF2* ablation in mouse liver tumors (*Nrf2* ^*−/−*^) leads to a global reduction in histone H3 acetylation on Lysine 27 (H3K27ac), marker of active enhancer regions (Xi et al., 2023), and as a consequence reduced enrichment of H3K27ac at representative genes involved in oxidative stress response (*i.e., Nqo1*), glycolysis (*i.e., Slc2a1*), TCA cycle (*i.e., Aco2*), indicating that *NRF2* activity sustains histone acetylation–dependent transcriptional programs. Similar findings have been reported in NSCLC, where NRF2 hyperactivation favours H3K27ac deposition (Okazaki et al., 2020). In this study, NSCLC-specific NRF2 target loci, that are absent in normal lung tissue, have been identified, suggesting a tumor-specific gene regulation. Notably, this regulation appears to involve the establishment of unique NRF2-dependent enhancer elements (Okazaki et al., 2020).

While chromatin remodelling may drive metabolic rewiring, metabolic reprogramming may also affect chromatin accessibility and therefore control gene expression (Lu and Thompson, 2012). This tight coupling has important implications for genome stability: NRF2 indeed may influence the recruitment and activity of DNA repair machinery, thereby linking metabolic and epigenetic regulation to DNA damage response, as described in the next paragraph. Understanding this connection provides a mechanistic framework to explore how NRF2 contributes to both cancer progression and resistance to genotoxic therapeutic approaches.

### 1.2 NRF2: DNA damage response and DNA repair

Genotoxic treatments induce NRF2, positioning it at the crucial intersection of metabolic adaptation and chromatin remodeling. This therapy-driven signaling dictates the accessibility of damaged DNA sites to repair factors, effectively connecting cellular defence with canonical DDR mechanisms. Importantly, by directly promoting the expression of several antioxidant genes such as *HMOX-1*, *NQO1* and *PRDX,* NRF2 ensures ROS scavenging and, by reflect, it alleviates the oxidative DNA damage in basal conditions as well as in response to chemotherapeutic treatments (Jiang et al., 2025; Jayakumar et al., 2015; Mirzaei et al., 2021; Li et al., 2023). Interestingly, the presence of AREs has been reported also within the promoter regions of many non-canonical target genes, even involved in the DNA damage response (DDR), spanning way beyond the traditional view of NRF2 as simply an antioxidant transcription factor (Li et al., 2023). Base Excision Repair (BER), one of the main pathways responsible for oxidative DNA damage repair, is in fact reported to be transcriptionally connected to NRF2 (Li et al., 2023). The presence of ARE sequences has been identified in the promoter regions of two players acting in the early stages of BER: eight-oxoguanine DNA glycosylase 1 (OGG1), responsible for 8-hydroxy-2′-deoxyguanosine (8-OHdG) removal from the DNA backbone (Singh et al., 2013), and MutY DNA glycosylase (MUTYH), able to remove the adenosine residues erroneously paired with 8-OHdG (Amirinejad et al., 2021). In this same pathway, by contrast, a negative feedback loop between NRF2 and flap structure-specific endonuclease 1 (FEN1) has been reported in MCF-7 cells. FEN1 is a protein involved in Okazaki fragments maturation and in the long patch BER (LP-BER), due to its ability to remove the DNA 5′-flap structures generated by this pathway (Chen et al., 2014). While the precise molecular mechanism driving this negative regulation remains poorly understood, one plausible explanation involves miR-140, a direct target of NRF2 (Duru et al., 2015) known to impair DDR by inhibiting FEN1 (Lu et al., 2020). However, the definitive existence of this specific signaling axis remains to be experimentally verified.

Other studies highlight a direct transcriptional role of NRF2 on *TP53BP1* and *BRCA1* genes, indicating an involvement of this transcription factor in both Non-Homologous End Joining (NHEJ) and Homologous Recombination Repair (HRR) pathways, respectively (Kim et al., 2012; Wang et al., 2013). Interestingly, BRCA1 has been reported to bind to NRF2’s Neh2 domain, competing with KEAP1 and thereby stabilizing NRF2 and enhancing its ARE-dependent transcription (Gorrini et al., 2013). Importantly, the expression of RAD51 is reduced upon NRF2 silencing and bioinformatic analysis revealed the presence of ARE sequences in the 5′ region of HR related genes, such as *RBBP8* (CtIP) and *RAD52* (Jayakumar et al., 2015). In addition, NRF2 plays a direct role in coordinating DDR through its interaction with checkpoint kinase pathways, particularly ATM-CHK2 and ATR-CHK1, creating a two-way regulatory network that connects oxidative stress management with genome stability (Sun et al., 2020; Jiang et al., 2025). Recent studies have revealed an unexpected side of this interaction: ROS act as signaling molecules and activate DDR pathways, which then regulate NRF2 stability and activity. During oxidative stress CHK2 kinase directly phosphorylates the autophagy adaptor protein p62 at serine-349 promoting p62 binding to KEAP1 and disrupting KEAP1-NRF2 complex, therefore preventing NRF2 degradation (Jiang et al., 2025). CHK2 also directly phosphorylates NRF2 itself at serine-566 and serine-577, increasing its transcriptional activity (Jiang et al., 2025). This dual mechanism, stabilizing NRF2 protein while boosting its activity, represents an efficient cellular strategy to coordinate antioxidant defence with DNA repair.

The relationship between NRF2 and the ATR-CHK1 pathway shows an even more complex regulatory network. NRF2 promotes radiation resistance in lung cancer by activating the ATR-CHK1 signaling pathway through TOPBP1 recruitment to DNA damage sites (Sun et al., 2024). Importantly, NRF2 supports ATR-mediated phosphorylation of RPA32, a key component of the replication protein A complex, independently of its transcriptional activity, as deletion mutants lacking the DNA-binding domain retain the ability to promote ATR-CHK1 phosphorylation following Ionizing Radiation (IR) (Sun et al., 2020). Mechanistically, NRF2 engages in direct protein-protein interactions at chromatin, enhancing TOPBP1 recruitment to RPA-coated ssDNA regions, thereby strengthening checkpoint signaling and promoting HRR (Sun et al., 2024). These findings have important clinical implications for cancer biology and therapeutic resistance. Galan-Cobo and colleagues recently showed that KRAS-mutant NSCLC with both KEAP1 and STK11/LKB1 mutations display unexpected sensitivity to ATR inhibition, despite their aggressive behaviour and resistance to standard therapies (Galan-Cobo et al., 2025). Mechanistically, sustained NRF2 activation caused by KEAP1 loss leads to increased ATR-CHK1 signaling, inducing a chronic replication stress state that could potentially be therapeutically exploited. This observation highlights a critical concept: the NRF2-DDR interaction operates within a delicate balance, where constitutive NRF2 activation, while initially protective, eventually makes cancer cells dependent on high DDR activity for survival, especially under conditions of elevated replication stress associated with LKB1 loss (Galan-Cobo et al., 2025).

Collectively, these findings reframe NRF2 not merely as a transcriptional antioxidant effector, but as an active coordinator of the cellular genotoxic stress response, whose dual function in redox regulation and DNA repair renders it indispensable to cancer cell survival under conditions of sustained oxidative damage.

### 1.3 NRF2: metabolism and inflammation

NRF2’s role in oxidative response and tumor metabolism concur to influence the tumor microenvironment (TME), including modulation of inflammation and immune system. NRF2 activity sustains glycolysis, glutathione synthesis, and NADPH production, that increase lactate levels in the extracellular microenvironment (Ricci, 2025).

Lactate acidifies the TME, thus reducing the activity and proliferation of CD8^+^ T cells (Fischer et al., 2007; Mendler et al., 2012; Fischbeck et al., 2020) and NK cells (Husain et al., 2013), while promoting immunosuppressive phenotypes in both myeloid (Myeloid-derived suppressor cells, MDSCs) (Colegio et al., 2014; Bohn et al., 2018; Zhang and Li, 2020) and lymphoid lineage (*e.g.*, Regulatory T cell, Treg) (Ricci, 2025; Chinen et al., 2016).

Concurrently, NRF2-dependent metabolic adaptations increase tumor consumption of key nutrients such as glucose, glutamine, and cysteine (Kiełbowski et al., 2025), creating metabolic competition which limits effector T-cell activity, thereby reinforcing tumor immune evasion and therapy resistance (Panda et al., 2025; Wen et al., 2025).

Moreover, by buffering ROS and antagonizing pro-inflammatory pathways such as NF-κB (Li et al., 2008) and STING (Olagnier et al., 2018), NRF2 significantly reduces the production of inflammatory factors which normally support antitumor immunity (Panda et al., 2025). Additionally, it directly sustains the expression of Programmed Death-Ligand 1 (PD-L1) (Payandeh et al., 2021) thereby inhibiting CD8^+^ T activation and affecting the response to immune checkpoint blockade therapy (Duan et al., 2023).

Of note, NRF2 is not only active in tumor cells but its expression is also induced in immune cell populations in response to different stimuli (Feng et al., 2023). Indeed, in response to oxidative stress or inflammation, NRF2 activation drives macrophage polarization toward an immunosuppressive phenotype M2-like (Feng et al., 2018; Wang and He, 2022), impairs dendritic cell maturation and antigen presentation, resulting in impaired T-cell priming, while sustains MDSCs (Panda et al., 2025). In T-cell, NRF2 plays mainly a cytoprotective role, but an excessive activation reduces effector functions and cytotoxic activity (Jo et al., 2016).

Moreover, NRF2 plays an important role in CD4^+^ T-cell activation, sustaining their metabolism through enhancing chromatin accessibility (Tripathi et al., 2025). Collectively, NRF2 mitigates stress and inflammation while promoting immunosuppressive phenotypes in the TME, largely through its role in buffering ROS. In most cancers, strong antitumor immunity is associated with high ROS levels and increased DNA damage, whereas immunosuppressive tumors limit ROS formation to preserve genomic integrity. NRF2 supports this immunosuppressive state by lowering ROS and protecting tumor cells from DNA damage, thereby contributing to resistance to genotoxic therapies (Tung et al., 2015; Yu et al., 2024; Guan et al., 2023).

However, GB presents a unique paradox: despite high baseline inflammation, its TME remains strongly immunosuppressive (Chen et al., 2025; Kruse et al., 2014), creating a permissive niche for tumor growth. In this context, NRF2 hyperactivation protects the DNA of tumor cells from ROS, drives antioxidant and metabolic programs, and reinforces immunosuppressive signaling. This enables GB cells to maintain genomic stability, survive inflammatory stress, and promote tumor progression and immune evasion.

### 1.4 NRF2 and drug resistance

Resistance to therapy remains a major challenge in oncology. Cancer cells can develop drug resistance before treatment (intrinsic resistance) or after treatment (acquired resistance) (Wang et al., 2019). Intrinsic resistance is usually driven by pre-existing mutations and tumor heterogeneity, including the presence of resistant cell subpopulations such as cancer stem cells (CSCs) that play a pivotal role in tumor initiation and progression (Chu et al., 2024). Acquired resistance develops after drug exposure through mechanisms including the activation of alternative oncogenic drivers, alterations in the drug target, and changes in the TME (Soragni et al., 2025). Together, these processes enable tumor cell survival and underlie the limited effectiveness of conventional therapies.

In this context, NRF2 plays a pivotal role across several cancer types, including GB, TNBC, and lung cancer, enhancing ROS scavenging and thereby strengthening resistance mechanisms that enable cancer cells to evade sensitivity to chemo- and radiotherapy (Verdina and D’Orazi, 2025; Panieri and Santoro, 2016; Xue et al., 2020) (Table 1).

**TABLE 1:** Schematic overview of tumors expressing high NRF2 levels and their response to different type of therapeutic treatments.

Tumor	Therapeutic treatment	Resistant/Sensitive	Mechanisms of therapy resistance	NRF2 driver(s)	References
Glioblastoma (GB)	Temozolomide (TMZ)-based therapy	Resistant	ROS scavenging, glycolysis, reduced DNA damage	Therapy-induced KEAP1 oxidation, p62 accumulation, and kinase PTMs	De Souza et al. (2022), Awuah et al. (2022), Tomar et al. (2026)
Glioblastoma (GB)	Radiotherapy	Resistant	Antioxidant response reduces radiation-induced ROS	Oncogenic kinase (Src) signaling, p62 accumulation, hypoxia, and metabolic reprogramming	Cirotti et al. (2024), Awuah et al. (2022), Tang et al. (2022), Ba et al. (2019)
Ovarian Cancer	PARP inhibitors	Resistant	Acetyl-CoA depletion, metabolic stress	NRF2 acetylation/epigenetic upregulation and oncogenic (RAS) signaling	Wilson et al. (2022), Basseville et al. (2022)
Head and neck squamous cell carcinomas (HNSCC)	Cisplatin (CDDP)-based chemotherapy	Resistant	ROS scavenging, detox pathways	Epigenetic reprogramming	Osm et al. (2023)
Head and neck squamous cell carcinomas (HNSCC)	Radiotherapy	Resistant	Reduced DNA damage	KEAP1/NRF2 mutations	Patel et al. (2025)
Triple-negative breast cancer (TNBC)	Chemotherapy (Paclitaxel, EGFR inhibitors)	Resistant	Detoxification enzymes, survival pathways	MET signaling, KEAP1 cysteinylation, and Akt crosstalk	Taddei et al. (2026), Bottoni et al. (2024), Kaboli et al. (2020)
Triple-negative breast cancer (TNBC)	Radiotherapy	Resistant	Reduced DNA damage	Therapy-induced ROS	Qin et al. (2021)
Breast cancer stem cells (BCSCs)	Radiotherapy	Resistant	Reduced DNA damage	Intrinsic stemness program and Akt signaling crosstalk	Qin et al. (2021), Canha-Borges et al. (2025)
Lung cancer (NSCLC/LUAD)	Chemotherapy/TKIs	Resistant	NRF2-driven antioxidant program, drug efflux	KEAP1/NRF2 mutations, CDK20 interaction, KRAS signaling, and therapy-induced adaptation	Jeong et al. (2020), Fouzder et al. (2024), Cescon et al. (2015), Wang et al. (2017), Tao et al. (2014), Krall et al. (2017)
LUAD	Immunotherapy	Resistant	Immune evasion	NRF2 hyperactivation, PI3K pathway synergy, and KEAP1 mutation	Almeida-Lima et al. (2025), Zavitsanou et al. (2023), Best et al. (2018)
LUAD	HDAC inhibitors (Romidepsin)	Sensitive	Metabolic vulnerability, ↓ NRF2 target genes, chromatin remodeling	KEAP1/NRF2 mutations	Karagiannis et al. (2024)

Several reports indicate the role of NRF2 in the maintaining of self-renewal capacity in CSCs linking its hyperactivation with intrinsic resistance (Choi et al., 2021). Of note, NRF2 knockdown reduces the proliferation and the expression levels of pluripotency-associated factors such as BMI-1, Sox2 and cyclin E both *in vivo* and *in vitro* glioma stem cells (GSCs) (Zhu et al., 2013). In addition, mammosphere cultures of breast cancer cells, commonly used to enrich for CSCs, exhibit reduced intracellular ROS levels due to NRF2 hyperactivation. This supports the maintenance of stem-like properties and contributes to both chemo- and radioresistance. Importantly, pharmacological NRF2 inhibition with Brusatol or ML-385 sensitizes mammospheres to paclitaxel (Taxol) or IR treatment, promoting the eradication of CSCs, suggesting a potential strategy to overcome therapy resistance (Wu et al., 2015; Qin et al., 2021).

As pointed out, prolonged drug exposure leads to acquired resistance. Both IR and chemotherapy induce NRF2 upregulation, contributing to cancer cells survival (Verdina and D’Orazi, 2025; Xue et al., 2020). Accordingly, NRF2 hyperactivation in HNSCC increases myeloid infiltration, angiogenic signatures accelerating tumour growth and contributing to radiotherapy resistance (Patel et al., 2025). Notably, NRF2-silenced GB cells exhibit elevated ROS levels and increased sensitivity to temozolomide (TMZ) treatment, along with increased levels of TMZ-induced DNA damage (Rocha et al., 2016). Moreover, we have recently demonstrated that NRF2 expression and activation are sustained by tyrosine kinases both in GB and TNBC and targeting these kinases enhances sensitivity to therapeutic approaches (Cirotti et al., 2024; Taddei et al., 2026). In particular, targeting the MET-SRC-NRF2 axis markedly enhances Paclitaxel sensitivity in both TNBC cell lines and patient-derived organoids (PDOs) revealing this signaling cascade as a therapeutically exploitable vulnerability in NRF2-hyperactivated TNBC (Taddei et al., 2026).

A key mechanism of therapy resistance involves NRF2-mediated upregulation of Multidrug Resistance Proteins (MRPs) acting as drug efflux transporters, which pump cytotoxic agents out of cells, thereby reducing their intracellular accumulation and limiting their toxicity (Bai et al., 2016). In this scenario, it has been demonstrated that NRF2-ARE pathway plays an essential role in regulating the expression of MRP1. Consistently, NRF2 knockdown reduces MRP1 expression, restoring the sensitivity to multiple chemotherapeutic agents in Small Cell Lung Carcinoma (SCLC) cells (Ji et al., 2013). Accordingly, the upregulation of MRP1 in GB is associated with increased tumor aggressiveness, chemotherapy resistance and poorer survival of patients. Moreover, NRF2 inhibition promotes ferroptotic cell death in GB cells, thereby enhancing their sensitivity to TMZ or IR treatment (Cirotti et al., 2024; De Souza et al., 2022).

In a recent study, Yanai and colleagues unveil the mechanisms activated by cancer cells to favour therapy resistance when exposed to increasing doses of the PARP inhibitor, Olaparib. They witnessed the appearance of several cell subpopulations defined “states”, each characterized by distinct expression signatures and by increased adaptability towards chemotherapy when transitioning from a former state to the next one. Notably, ATAC-seq analyses revealed that the most resistant states present an increase in chromatin accessibility for several stress regulators, including NRF2 (França et al., 2024).

As chromatin remodelling represent a critical issue for cancer treatment, so far HDAC inhibitors are in clinical trials showing that their activity is enhanced when combined with other anticancer agents (Abdallah, 2026). Among these, romidepsin and vorinostat, are FDA-approved drugs currently used for treatment of refractory T cell lymphoma (Mann et al., 2007; Iyer and Foss, 2015; Karagiannis et al., 2024) as well as DNA methyltransferase inhibitors for myeloid malignancies (Diesch et al., 2016). On the contrary their use in solid tumours is still under investigation. In this regard, using a chromatin-focused CRISPR screen, it has been recently demonstrated that lung adenocarcinoma (LUAD) having high NRF2 activity is highly sensitive to class I HDAC inhibitors. These inhibitors strongly affect amino acid and nucleotide metabolism, thus decreasing cancer cell aggressiveness and proliferation. Specifically, Romidepsin is particularly toxic to NRF2-activated cells because it represses NRF2-driven metabolic genes. This repression alters chromatin structure and increases NRF2-dependent metabolic vulnerabilities, contributing to cell toxicity (Karagiannis et al., 2024).

## 2 Conclusion

**FIGURE 1:**
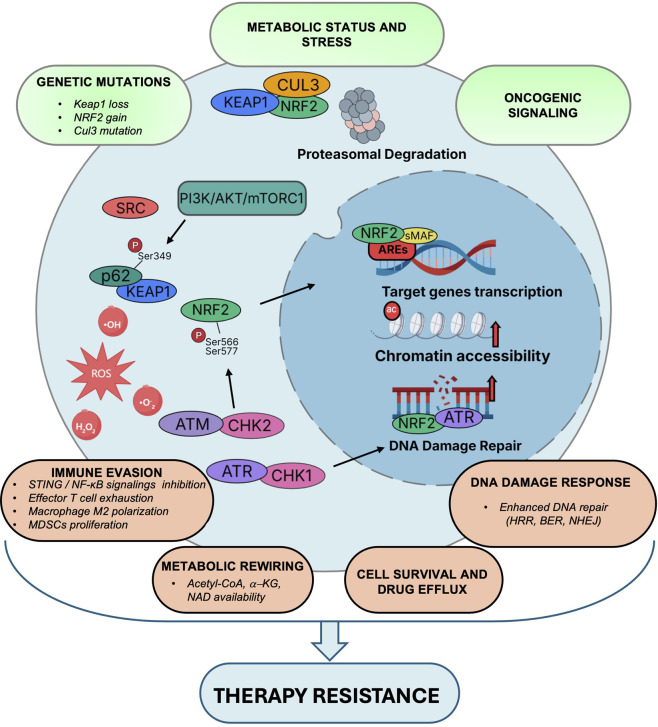
Wiring NRF2 for Tumor Survival and Resistance. Top panels (green) depict the diverse upstream inputs including oxidative stress, aberrant oncogenic signaling, and genetic alterations that drive NRF2 stabilization. Bottom panels (orange) outline the downstream effector functions of the NRF2 network. Upon stabilization, NRF2 orchestrates a comprehensive cytoprotective response. By coordinating DNA damage repair, reprogramming cellular metabolism, and altering chromatin accessibility, NRF2 aberrant signaling drives tumor malignancy and confers resistance to therapeutic interventions. Figure created with BioSketch.art.

NRF2 hyperactivation is frequently observed in tumors, where it correlates with poor patient survival. Through modulation of cellular metabolism and gene expression, NRF2 impairs genotoxic therapy efficacy by influencing the DNA damage response and suppressing anti-tumor immunity, driving therapy resistance (Figure 1). However, direct pharmacological targeting of NRF2 remains challenging due to the lack of inhibitors that efficiently target its activity. Several studies have identified NRF2 inhibitors including small molecules and natural plant-derived compounds; unfortunately, none have been successful in clinical trials. In this regard, there is an urgent need to identify novel targetable co-players that cooperate with NRF2 in promoting tumor aggressiveness. Assessing NRF2 status alongside these molecular players may therefore guide more effective therapeutic decisions and support the development of alternative combination strategies.
